# Letter from the Editor in Chief

**DOI:** 10.19102/icrm.2024.15116

**Published:** 2024-11-15

**Authors:** Devi Nair


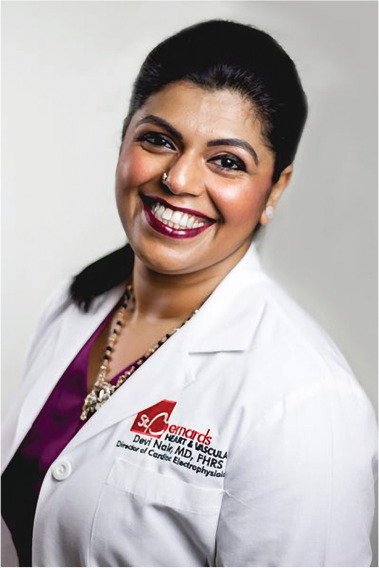


Dear Readers,

As we welcome November, it’s truly remarkable to reflect on the swift progress in the field of cardiac rhythm management, especially when it comes to the advancements and adoption of pulsed-field ablation (PFA). This month, we saw the U.S. Food and Drug Administration approve two new PFA systems, Affera (Medtronic, Minneapolis, MN, USA) and Varipulse (Biosense Webster, Diamond Bar, CA, USA), further expanding our toolkit for treating atrial fibrillation (AF) with precision and efficiency. These recent approvals signify not only a deepening validation of PFA but also underscore its potential to redefine how we approach catheter ablation by offering tissue selectivity, safety, and procedural efficiency. The rapid adoption of PFA across the United States, with more centers incorporating PFA, speaks to the broader trend within electrophysiology: an eagerness to adopt and refine technologies, and our field’s readiness to embrace this promising technology. It will be exciting to observe its impact on clinical practice and patient care, as well as on our understanding of lesion formation and durability over time.

However, as we celebrate these advances, we must remain cautiously optimistic. While PFA’s safety profile is promising, particularly in terms of minimizing collateral damage to surrounding structures, we are aware of potential risks that warrant close monitoring. As adoption grows, it will be critical to monitor for any issues related to hemolysis, coronary spasm, and possible progression of coronary artery disease. These considerations are essential to ensuring that PFA lives up to its potential as a safe and effective option for AF treatment without introducing unintended complications.

Another transformative development this month is the approval of a dedicated Diagnosis-related Group code for concomitant AF ablation and left atrial appendage closure (LAAC) procedures. This addition opens the door for clinicians to more seamlessly integrate ablation with LAAC, addressing both rhythm control and stroke risk in a single procedural setting for the indicated patients. This new code not only provides a clearer path for reimbursement but also recognizes and acknowledges the importance of comprehensive AF management with a growing emphasis on personalized, combined therapeutic strategies in AF care. However, it also raises critical questions: how will the availability of this combined approach shape clinical decision-making and treatment paradigms? Will it lead us to reconsider how we assess risk stratification and determine the best course of action for individual patients? As we delve into these questions, it is imperative that we also continue gathering data on the long-term outcomes and cost-effectiveness of these concomitant procedures.

This month’s issue of *The Journal of Innovations in Cardiac Rhythm Management* features a collection of articles that showcase the depth and breadth of innovation in cardiac rhythm management. From studies on the latest ablation techniques to explorations of integrative approaches like LAAC, we are committed to fostering a platform where critical advancements and emerging insights can reach the global community. As we navigate this evolving landscape, *JICRM* remains dedicated to supporting the dissemination of knowledge and dialogue that ultimately drives our field forward.

Thank you to all our authors, reviewers, and readers for your commitment and contributions. Together, we are shaping a future where our tools and techniques better serve our patients and deepen our understanding of complex cardiac arrhythmias. I look forward to seeing how these recent developments will inspire new directions in our pursuit of more effective, patient-centered care.

Warm regards,


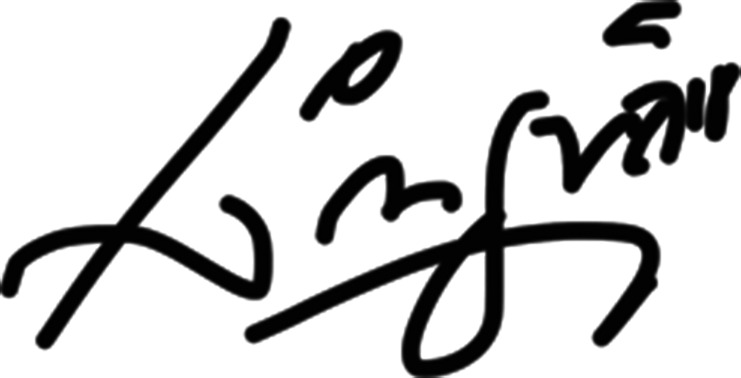


Dr. Devi Nair, md, facc, fhrs

Editor-in-Chief

*The Journal of Innovations in Cardiac Rhythm Management*

Director of the Cardiac Electrophysiology & Research,

St. Bernard’s Heart & Vascular Center, Jonesboro, AR, USA

White River Medical Center, Batesville, AR, USA

President/CEO, Arrhythmia Research Group

Clinical Adjunct Professor, University of Arkansas for Medical Sciences

Governor, Arkansas Chapter of American College of Cardiology

drdgnair@gmail.com

